# The burden of HIV among female sex workers, men who have sex with men and transgender women in Haiti: results from the 2016 Priorities for Local AIDS Control Efforts (PLACE) study

**DOI:** 10.1002/jia2.25281

**Published:** 2019-07-09

**Authors:** Lauren C Zalla, Michael E Herce, Jessie K Edwards, Jacob Michel, Sharon S Weir

**Affiliations:** ^1^ Department of Epidemiology University of North Carolina at Chapel Hill Chapel Hill NC USA; ^2^ Carolina Population Center University of North Carolina at Chapel Hill Chapel Hill NC USA; ^3^ Division of Infectious Diseases Department of Medicine University of North Carolina at Chapel Hill Chapel Hill NC USA; ^4^ FHI 360 Port‐au‐Prince Haiti

**Keywords:** transgender people, sex workers, men who have sex with men, viral suppression, Haiti, Caribbean

## Abstract

**Introduction:**

Despite the higher risk of HIV among female sex workers (FSWs), men who have sex with men (MSM) and transgender women (TGW), these populations are under‐represented in the literature on HIV in Haiti. Here, we present the first nationally representative estimates of HIV prevalence and the first care and treatment cascade for FSWs, MSM and TGW in Haiti. We also examine the social determinants of HIV prevalence in these groups and estimate FSW and MSM population size in Haiti.

**Methods:**

Data were collected between April 2016 and February 2017 throughout the 10 geographical departments of Haiti. The Priorities for Local AIDS Control Efforts (PLACE) method was used to: (1) recruit participants for a behavioural survey; (2) provide rapid testing, counselling and linkage to care for syphilis and HIV; and (3) measure viral load using dried blood spots for participants testing HIV positive.

**Results:**

Study participants included 990 FSWs, 520 MSM and 109 TGW. HIV prevalence was estimated at 7.7% (95% CI 6.2%, 9.6%) among FSWs, 2.2% (0.9%, 5.3%) among MSM and 27.6% (5.0%, 73.5%) among TGW. Of participants who tested positive for syphilis, 17% of FSWs, 19% of MSM and 74% of TGW were co‐infected with HIV. Economic instability and intimate partner violence (IPV) were significantly associated with HIV among MSM; food insecurity, economic instability and history of rape were significantly associated with HIV among TGW. Fewer than one‐third of participants living with HIV knew their status, and more than a quarter of those who knew their status were not on treatment. While approximately four in five FSW and MSM participants on treatment for HIV were virally suppressed, viral suppression was less common among TGW participants at only 46%.

**Conclusions:**

This study demonstrates a need for targeted interventions to prevent and treat HIV among key populations in Haiti. Potential high‐impact interventions may include venue‐based, peer navigator‐led outreach and testing for HIV and syphilis and improving screening and case management for structural violence and IPV. TGW are in urgent need of such interventions due to our observations of alarmingly high HIV prevalence and low frequency of HIV viral suppression among TGW.

## 1 Introduction

Since 2000, HIV prevalence has remained relatively stable in Haiti 1, with the most recent data suggesting an HIV prevalence of 2.0% among adults ages 15 to 49 2. The country continues to make progress towards controlling the epidemic and expanding access to antiretroviral therapy (ART) 3. Yet, its progress will be limited as long as unmet needs remain among key populations (KP) disproportionately affected by HIV, including men who have sex with men (MSM), female sex workers (FSWs) and transgender women (TGW).

Studies throughout the Caribbean document disproportionately high HIV prevalence among KP. For example, HIV prevalence estimates among MSM range from 6.7% in Suriname and 11% in the Dominican Republic to as high as 32% in Jamaica 4. Reports of HIV prevalence among FSWs range from 5% in the Dominican Republic and Jamaica to as high as 24% in Suriname 4. In Haiti, KP are not yet adequately represented in the literature on HIV. Of particular concern is the lack of data on virologic suppression among KP on ART in Haiti, which is necessary to measure progress against UNAIDS 90‐90‐90 targets 5. Another gap is the lack of national and subnational‐level size estimates for KP, which are critical to inform national strategy around resource allocation, intervention planning and programme evaluation 6.

In addition, there is limited recent biobehavioural survey (BBS) data to contextualize the complex interplay among social and structural factors driving the HIV epidemic among KP in Haiti. Previous studies have focused primarily on biomedical and behavioural risk factors such as condom use and alcohol consumption. For example, a 2015 study reported that 72.7% of MSM used a condom during their last sexual encounter with a man and 89.1% of FSWs used a condom during their last sexual encounter with a client 7, 8. While recent studies have considered some interpersonal factors such as intimate partner violence (IPV), very little is known about the broader social and structural risk factors for HIV acquisition among KP in Haiti.

We address these gaps with data from a 2016 Priorities for Local AIDS Control Efforts (PLACE) study designed to provide a robust epidemiologic profile of HIV among KP in Haiti, with the following specific objectives: (1) estimate the population size of FSWs and MSM in Haiti; (2) estimate the population prevalence of HIV among FSWs, MSM and TGW; (3) describe the underlying vulnerabilities and behavioural factors associated with HIV in KP; and (4) describe the HIV care and treatment cascade among KP.

## 2 Methods

The PLACE method is designed to involve local stakeholders in collecting data to inform the HIV epidemic response. PLACE systematically identifies and maps public venues where people meet new sexual partners, and then uses these maps as a sampling frame to reach, interview and test populations at risk of HIV 9, 10, 11, 12. UNAIDS recommends PLACE for biobehavioural surveys and KP size estimates 13.

Beginning in November 2015, we engaged stakeholders including policymakers, health providers and KP to discuss protocol issues, including terminology, access to KP, safety concerns and criteria for selecting study geographical areas. Each of the 42 *arrondissements* was assigned a priority score based on subnational estimates of HIV prevalence 14 and local contextual factors that stakeholders believed to be associated with the KP population. Contextual factors such as ports, major roads, tourist attractions, nightlife and vodou peristyles were used as proxy indicators for KP size, since no subnational size estimates were available prior to this study. A stratified random sampling approach was undertaken to ensure a nationally representative sample of venues and participants while maximizing the inclusion of KP. We selected 100% of 8 high‐priority, 90% of 16 medium‐priority and 10% of 18 low‐priority *arrondissements*, resulting in 24 *arrondissements* selected for PLACE. Before data collection began, we met with the local health department in each study area as well as local civil society organizations serving KP.

During Phase I of data collection, interviewers captured an exhaustive list of venues in sampled *arrondissements* by canvassing a sufficiently large and diverse pool of knowledgeable community informants. Each sampled *arrondisement* was divided into smaller zones of 50,000 inhabitants based on the latest census data 15. A minimum of 30 community informants was interviewed in each zone. To ensure a diverse pool of informants, supervisors assigned each interviewer daily targets for the number and type of informants interviewed. Examples of types of informants included police, street vendors, bartenders, moto‐taxi drivers, security guards, hairdressers, community leaders, peer educators and KP.

During Phase II, interviewers attempted to visit all venues identified in Phase I. Venues were classified as found and operational, not found, closed temporarily, closed permanently or duplicates of other venues. At all venues that were found and operational, interviewers validated the information provided by community informants by interviewing a venue manager, owner or regular patron. The information collected included operating hours and busy times, activities and amenities, the availability of on‐site prevention services such as HIV testing, peer education and condom distribution, and the number of venue patrons including KP.

During Phase III, a BBS was conducted at two stratified random samples of validated venues. The first sample oversampled venues where MSM were reported during Phase II: 100% of venues with ≥10% MSM relative to all male patrons, 20% with <10% MSM and 1% with no MSM. At these venues, all men were approached for an interview. The second sample oversampled venues reported to be frequented by FSW: 80% of venues with ≥10% FSW relative to all female patrons, 5% with <10% FSW and 1% with no FSW. At these venues, all women were approached. Transgender people could be approached at either type of venue. Every venue had a known and non‐zero probability of being selected and the same venue could be selected by chance into both samples. The total number of venues selected into each sample was chosen to facilitate the recruitment of ≥600 FSWs and ≥600 MSM based on the venue‐level estimates collected in Phase II.

Interviewers and nurses visited each sampled venue at a busy time and set up testing tables in private, quiet locations. Eligible participants were individuals aged ≥15 years who responded positively to a single screening question, designed by stakeholders to be non‐stigmatizing, which asked whether the respondent engaged in any of the following in the past three months: had sex with ≥3 different persons, had anal sex with anyone or met a new sexual partner at a public festival. The question aimed to ensure inclusion of all MSM and FSW without requiring self‐identification as KP.

The interview included questions about demographic characteristics, venue‐visiting behaviour, sexual behaviour, sexual health and access to services. Participants were also screened for social vulnerabilities experienced in the past year, such as food insecurity (not always having enough food to eat), economic insecurity (not always having enough money to support oneself), homelessness (not always having a place to sleep) and IPV (experiencing physical violence inflicted by a sexual partner). Some questions asked about lifetime social vulnerabilities such as rape (ever forced to have sex against one's will or without a condom), imprisonment (ever spending a night in jail or prison) and police violence (ever beaten by a police officer).

### 2.1 Clinical and laboratory procedures

All participants received pre‐ and post‐test HIV counselling from a trained nurse counsellor. Nurses also administered interview questions about HIV status and other sexually transmitted infections (STIs), based on stakeholder feedback that participants would feel more comfortable sharing this information with a health professional. Following national guidelines 16, nurses administered rapid tests for syphilis and HIV to all participants using SD Bioline Syphilis 3.0 and Alere Determine™ HIV‐1/2 (Abbott Laboratories, Abbott Park, IL, USA). Positive HIV tests were confirmed using either OraQuick (OraSure Technologies, Inc., Bethlehem, PA, USA) or UniGold (Trinity Biotech, Bray, Co. Wicklow, Ireland). Participants with a positive or indeterminate HIV test result were referred for follow‐up testing and treatment at a government health facility, and a peer navigator was assigned to facilitate linkage to care.

Dried blood spot (DBS) samples were collected from participants with positive and indeterminate test results for viral load (VL) testing at the University of North Carolina–Chapel Hill (UNC‐CH) 17. DBS samples were stored and transported in ziplock bags with desiccation packets according to established methods 17. Two whole blood spots were taken from each card, eluted in RNA lysis buffer (Promega Corporation, Madison, WI, USA) and run on the Abbott RealTime HIV‐1 viral load assay (Abbott Molecular, Des Plaines, IL, USA). Viral suppression was defined based on the assay's lower limit of detection (1360 copies of HIV‐1/mL).

### 2.2 Analytical methods

#### 2.2.1 Specific objective 1

The crude size estimate (SE) for an *arrondisement* was calculated by summing the number of FSW or MSM present at a venue at a busy time, as reported by the venue informant, across all venues in the *arrondisement*. Crude SEs were adjusted using counts of KP at a probability‐based sample of venues visited for Phase III. SEs were also adjusted using data from individual interviews to account for venue‐visiting behaviours, such as visiting multiple sites in one day. We extrapolated the adjusted SEs to *arrondissements* we did not visit using Poisson regression 6. We did not calculate SEs for TGW since most venue informants could not accurately identify TGW.

#### 2.2.2 Specific objective 2

To estimate the prevalence of HIV by KP, we applied survey sampling weights in Stata (Version 14.2, StataCorp LLC, College Station, TX), reweighting study participants to represent the broader populations of FSW, MSM and TGW. Standard errors were corrected for the stratified sampling approach and the clustering of individuals within venues and venues within *arrondissements*. We accounted for missing data using inverse probability of selection weights based on predicted probabilities of refusal conditional on venue type and *arrondisement* 18.

#### 2.2.3 Specific objective 3

We used log‐binomial regression to estimate weighted bivariate associations between various exposures and HIV.

#### 2.2.4 Specific objective 4

We present the number of HIV‐positive participants by KP at each step of the treatment cascade.

### 2.3 Ethical considerations

The study protocol was reviewed and approved by the Comité National de Bioéthique d'Haïti and the UNC‐CH Institutional Review Board (Protocol #15‐3242). Informed consent was administered to all individuals screened as eligible. A waiver of parental consent was granted for participants ages 15 to 17. Following stakeholder advice, no written documentation of test results was provided as FSWs are often forced to disclose results to managers and clients. All completed surveys were immediately encrypted and stored on a secure server.

## 3 Results

### 3.1 Fieldwork summary

A total of 5032 community informants were interviewed in Phase I from April to May 2016. Interviewers compiled a list of over 3500 venues mentioned by community informants, of which 2339 were unique venues that were found and operational in June to July 2016. Of the venues that were not found and operational, 18% were not found, 10% were closed temporarily, 11% were closed permanently and 61% were duplicates. Characteristics of the validated venues are reported in Table S1. One‐third of venues had male condoms available on site in the past six months, 15% had been visited by an outreach worker and 10% had on‐site HIV testing services (HTS).

**Figure 1:**
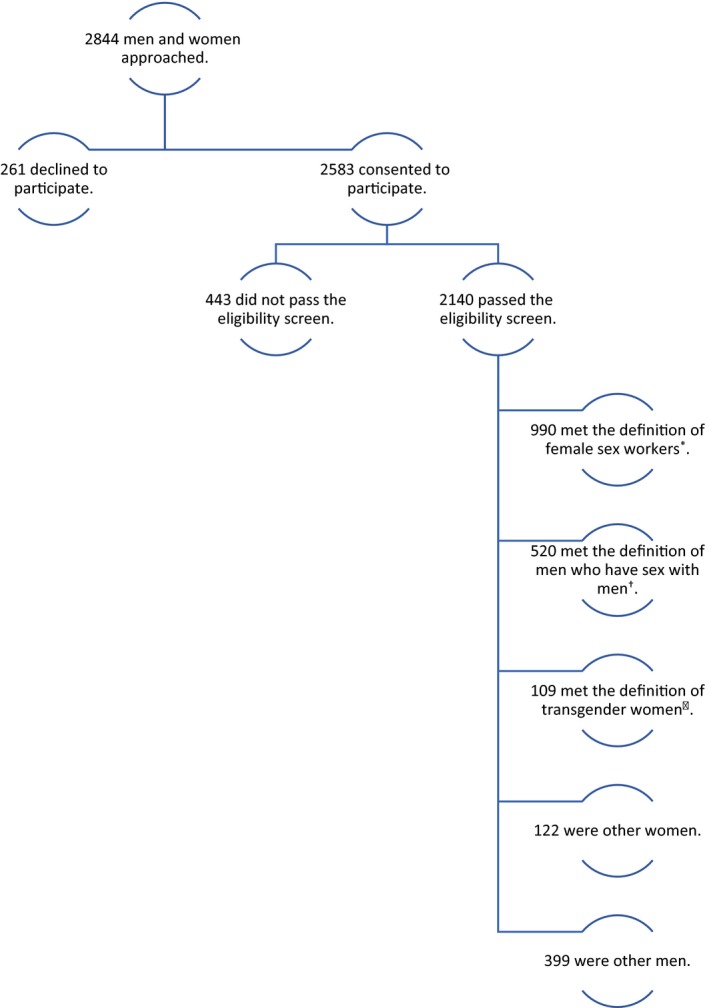
Participant flow diagram, Phase III, Priorities for Local AIDS Control Efforts 2016. *Female sex workers (FSWs) were participants who reported female sex at birth and receiving any money, gifts or favours in exchange for sex in the past year 36. ^†^Men who have sex with men (MSM) were participants who reported male sex at birth, male gender identity and having any male sexual partners in the past year. ^‡^Transgender women (TGW) were participants who reported male sex at birth and female gender identity.

During Phase III, 2844 men and women were invited to participate in interviews and to receive HTS at 268 sampled venues between August 2016 and February 2017. Of the 2583 individuals who consented to participate, 2140 (83%) passed the eligibility screen and completed interviews and testing (Figure 1).

### 3.2 Female sex workers

We estimate that 40,400 FSWs can be reached at venues where people meet new sexual partners in Haiti, representing 1.5% of the female population ages 15 to 49.

Of the 1112 female study participants, 990 reported receiving money or gifts in exchange for sex in the past year. Compared to female participants who had not engaged in sex work in the past year, those who had tended to be older, single, have a lower level of education and have dependent children (Table 1). More than half of FSWs had ever been raped and nearly one‐third had experienced IPV in the past year (Table 2). Over 16% of FSWs did not receive any condoms in the past year; half of FSWs who reported having anal sex received lubricant. While 80% of FSWs knew where they could get an HIV test, half of those (42%) had been tested in the past three months and 60% of FSWs of negative or unknown HIV status perceived themselves to be at no or low risk of acquiring HIV.

**Table 1:** Demographic characteristics of participants by study population and weighted estimates of HIV and syphilis prevalence, PLACE 2016

	FSWs	MSM	TGW	Other women	Other men
N	990	520	109	122	399
Median age (IQR)	26 (23 to 31)	21 (19 to 25)	24 (21 to 29)	23 (19 to 27)	24 (21 to 30)
Per cent with any source of income	94.3	54.8	48.6	55.7	67.2
Level of education					
None	5.6	1.2	0.9	0.8	1.0
Primary	22.8	12.7	7.3	11.5	11.3
Secondary or higher	71.6	86.2	91.7	87.7	87.7
Per cent in a primary relationship	49.5	70.8	63.3	65.6	66.9
Median number of dependent children (IQR)	1 (1 to 2)	0 (0 to 1)	0 (0 to 1)	1 (0 to 1)	0 (0 to 1)
Median age of sexual debut (IQR)	16 (14 to 17)	14 (11 to 16)	13 (10 to 15)	16 (14 to 17)	13 (11 to 15)
Per cent residing in same commune where interviewed	58.3	92.8	84.8	68.5	89.3
Per cent who self‐identify as LGBTQ	5.6	63.7	89.0	2.5	4.5
Estimated HIV prevalence (95% CI)	7.7% (6.2, 9.6)	2.2% (0.9, 5.3)	27.6% (5.0, 73.5)	0.7% (0.1, 5.0)	1.1% (0.4, 3.4)
Estimated syphilis prevalence (95% CI)	16.4% (12.4, 21.5)	4.2% (1.4, 11.8)	33.4% (11.0, 67.3)	2.9% (0.8, 9.7)	9.2% (3.5, 22.0)

FSWs, female sex workers; MSM, men who have sex with men; PLACE, Priorities for Local AIDS Control Efforts; TGW, transgender women.

**Table 2:** Population estimates of individual‐ and structural‐level risk factors for HIV among FSWs, MSM and TGW estimated from a representative survey of 990 FSWs, 520 MSM and 109 TGW in Haiti, PLACE 2016

	FSWs		MSM		TGW	
	Median	IQR	Median	IQR	Median	IQR
Individual behaviours						
No. of male partners in past four weeks	9	5 to 30	1	1 to 2	5	2 to 15
No. of female partners in past four weeks	0	0 to 2	3	2 to 5	3	2 to 5
	Per cent	95% CI	Per cent	95% CI	Per cent	95% CI
Ever had insertive or receptive anal sex	9.5	6.5 to 13.7	98.5	92.0 to 99.7	92.3	55.8 to 44.2
Received money or gifts for sex in past 12 months	100		64.5	31.1 to 88.0	82.3	66.4 to 91.6
Paid others money or gifts for sex in past 12 months	0.7	0.2 to 2.5	71.4	52.9 to 84.7	45.8	23.0 to 70.5
Used a condom at last vaginal sex	61.5	29.1 to 86.2	69.0	49.4 to 83.5	77.8	58.2 to 89.8
Used a condom at last anal sex	19.3	7.0 to 42.9	74.3	44.7 to 91.2	81.3	58.0 to 93.2
Used lubricant at last anal sex	6.2	1.4 to 23.7	42.5	18.6 to 70.4	72.8	43.9 to 90.1
Knowledge and perceived risk						
Correctly identified mode of transmission of HIV	63.8	55.6 to 71.2	68.4	41.7 to 86.7	59.8	36.9 to 79.1
Know where to get an HIV test	79.2	60.2 to 90.6	83.6	62.0 to 94.1	92.0	82.1 to 96.7
Perceived risk of acquiring HIV						
None	23.2	18.1 to 29.3	41.1	30.0 to 53.1	24.4	12.6 to 41.9
Low	36.7	25.0 to 50.3	25.9	9.3 to 54.4	45.5	24.9 to 67.8
Moderate	19.6	16.2 to 23.4	20.3	8.9 to 39.7	18.9	7.8 to 39.3
High	7.0	3.1 to 15.1	4.6	2.0 to 10.5	1.7	0.4 to 6.4
Do not know	13.5	5.5 to 29.6	8.2	4.3 to 15.0	9.5	1.1 to 49.9
Structural violence						
Not always able to pay basic expenses in past 12 months	50.3	29.7 to 70.8	57.3	28.2 to 82.1	48.9	29.2 to 69.0
Did not always have enough to eat in past 12 months	38.3	25.0 to 53.7	40.4	31.9 to 49.6	32.1	12.1 to 61.9
Ever homeless in past 12 months	29.7	25.4 to 34.4	15.6	5.1 to 38.5	23.7	12.3 to 40.7
Ever imprisoned	13.0	4.9 to 30.0	15.1	4.6 to 39.2	16.6	6.2 to 37.5
Interpersonal violence						
Ever forced to have sex against will or without a condom	54.8	46.8 to 62.6	39.6	17.5 to 67.1	76.5	50.9 to 91.1
Ever beaten by a police officer	7.5	2.4 to 20.7	5.6	1.6 to 17.4	9.2	1.2 to 45.9
Victim of intimate partner violence in past 12 months	31.5	24.5 to 39.6	15.7	5.4 to 37.7	26.0	6.1 to 65.5
Access to services						
Received free condoms in past 12 months	83.5	79.0 to 87.2	95.5	89.0 to 98.2	83.9	65.1 to 93.6
Received free lubricants in past 12 months	26.2	10.8 to 50.9	48.6	23.0 to 75.0	75.2	49.1 to 90.5
Use a method of contraception other than condoms						
None	49.1	33.0 to 65.5	N/A		N/A	
Oral contraceptive	3.3	1.0 to 10.7				
Injection	42.5	37.0 to 48.2				
Implant	4.4	0.4 to 33.0				
Other	0.6	0.1 to 3.3				
Ever tested for HIV	79.1	64.2 to 88.9	85.1	74.9 to 91.6	99.1	96.2 to 99.8
Tested for HIV in past 3 months	42.3	27.0 to 59.2	48.1	21.9 to 75.4	61.9	29.8 to 86.2

FSWs, female sex workers; MSM, men who have sex with men; PLACE, Priorities for Local AIDS Control Efforts; TGW, transgender women

**Figure 2:**
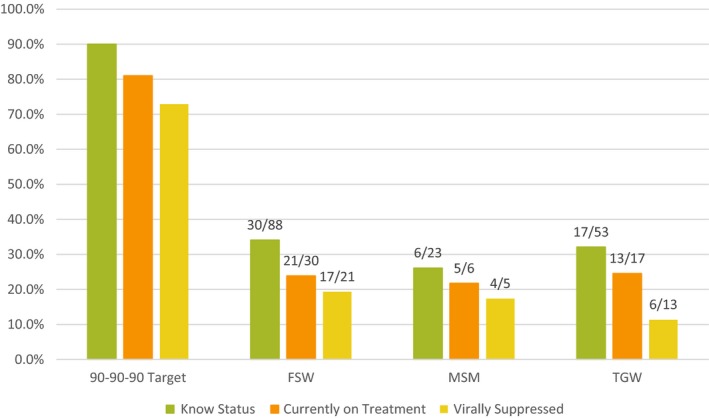
Number of participants with HIV at each step of the treatment cascade by key populations, PLACE, Priorities for Local AIDS Control Efforts 2016.

HIV prevalence was 7.7% (95% CI 6.2%, 9.6%) among FSWs. Syphilis prevalence was estimated at 16.4% (95% CI 12.4%, 21.5%), and 17% of FSWs with syphilis antibodies were HIV co‐infected. Two‐thirds of HIV‐positive FSWs did not know their status (Figure 2). Of those currently on ART, 81% had a suppressed VL. Food and economic insecurity, imprisonment, rape and IPV were positively but not significantly associated with HIV infection among FSWs (Table 3). Homelessness initially appeared to be protective among FSW, but after controlling for educational attainment and economic instability the PR was only marginally statistically significant at 0.28 (95% CI 0.08, 1.03).

**Table 3:** Prevalence ratios (PR) and 95% confidence intervals (CI) for the total associations between social determinants of health and prevalence of HIV by KP, PLACE 2016

	MSM (n = 488)		FSWs (n *= *958)		TGW (n *= *104)	
	PR	95% CI	PR	95% CI	PR	95% CI
Did not always have enough to eat in past 12 months	13.70	3.90, 48.10	1.38	0.53, 3.58	5.23	1.33, 20.52
Not always able to pay basic expenses in past 12 months	41.73	11.21, 155.36	1.92	0.84, 4.41	7.31	2.62, 20.39
Ever homeless in past 12 months	0.04	0.01, 0.35	0.27	0.07, 0.96	1.71	0.34, 8.65
Victim of intimate partner violence in past 12 months	51.58	20.47, 129.98	3.72	0.36, 38.21	2.33	0.23, 23.08
Ever imprisoned	0.58	0.14, 2.43	1.59	0.66, 3.87	0.44	0.11, 1.76
Ever forced to have sex against will or without a condom	8.19	3.09, 21.67	2.07	0.32, 13.45	7.02	1.90, 26.00

FSWs, female sex workers; KP, key populations; MSM, men who have sex with men; PLACE, Priorities for Local AIDS Control Efforts; TGW, transgender women.

### 3.3 Men who have sex with men

We estimate that 38,300 MSM can be reached at venues where people meet new sexual partners in Haiti, representing 1.4% of the male population ages 15 to 49.

Of the 919 men interviewed, 520 reported having sex with men in the past year; they were slightly younger and less likely to have a source of income than male participants who were not MSM (Table 1). Forty per cent of MSM had ever been raped and 16% had experienced IPV in the past year (Table 2). Nearly all MSM had received condoms at least once in the past year, and condom use at last vaginal and anal sex, respectively, was 69% and 74%. About half (49%) of MSM had received lubricant in the past year, and 43% used lubricant at last anal sex. While 84% of MSM knew where they could get an HIV test, half of those (48%) had been tested in the past three months, and 67% of MSM of negative or unknown HIV status perceived themselves to be at no or low risk of acquiring HIV.

HIV prevalence was 2.2% (95% CI 0.9%, 5.3%) among MSM. Nearly three‐quarters (73.9%) of HIV‐positive MSM did not know their status (Figure 2). Syphilis prevalence was estimated at 4.2% (95% CI 1.4%, 11.8%) and 19% of MSM with syphilis antibodies were HIV co‐infected. Of those currently on ART, 80% had a suppressed VL. HIV prevalence among MSM who suffered economic instability was 42 times the prevalence among those who were able to pay basic expenses (95% CI 11.2, 155.4), and MSM who had suffered IPV had over 50 times the prevalence of MSM who did not experience IPV (95% CI 20.5, 130.0). Homelessness initially appeared to be protective among MSM, but after controlling for educational attainment and economic instability the PR for the association between homelessness and HIV was 0.31 (95% CI 0.03, 3.45).

### 3.4 Transgender women

We interviewed 109 TGW. Compared to MSM, these participants were slightly older, slightly less likely to have a source of income or be in a primary relationship, and more likely to self‐identify as LGBTQ (Table 1). Over three‐quarters of TGW had been raped in their lifetime. Nearly a quarter were homeless and 17% were imprisoned in the past year. Over 90% of TGW knew where to get an HIV test, but less than two‐thirds had been tested in the past three months, and 70% of TGW of negative or unknown HIV status perceived themselves to be at no risk or low risk of acquiring HIV.

TGW had the highest HIV prevalence of any group at 27.6% (95% CI 5.0%, 73.5%). In fact, in every age group over age 25, >50% of TGW tested positive for HIV (Table sS2). Two‐thirds (68%) of HIV‐positive TGW did not know their status (Figure 2). Less than half (46%) of all TGW currently on ART were virally suppressed. Syphilis prevalence was estimated at 33.4% among TGW (95% CI 11.0%, 67.3%) and 74% of those with syphilis antibodies were HIV co‐infected. Food insecurity (PR 5.2, 95% CI 1.3, 20.5), economic instability (PR 7.3, 95% CI 2.6, 20.4) and rape (PR 7.0, 95% CI 1.9, 26.0) were all strongly associated with HIV infection among TGW.

## 4 Discussion

This study contributes the first nationally representative estimates of HIV prevalence and associated risk factors among KP in Haiti. This is also the first report of HIV prevalence among Haitian TGW, who had the highest HIV prevalence of all study groups at 28%. For FSW, estimated HIV prevalence was 5.4% points higher than the most recent estimate for women ages 15 to 49, and for MSM, we observed an HIV prevalence 0.6% points higher than that reported among all men ages 15 to 49 2. The elevated HIV prevalence observed among KP may be partly explained by pervasive structural violence, including poverty, homelessness and food insecurity. Our findings underscore the need for greater investment in efforts to realize the first 90 for KP in Haiti and to mitigate HIV risk through structural and other interventions designed to improve HIV prevention, treatment and care for KP.

Previous HIV surveillance studies from Haiti have grouped TGW together with MSM 7, 19. This is a common practice in the Caribbean 20, 21 despite acknowledgement that TGW may have different characteristics than MSM 22, 23. Despite our small sample of TGW, our findings suggest that HIV prevalence may be much higher among TGW than MSM.

We also observed that 4.2% of non‐KP men and 2.6% of non‐KP women socializing at venues who passed our eligibility screen tested HIV positive, which is higher than the most recent gender‐disaggregated general population estimates 2. Of 399 non‐KP men, 241 (60%) reported paying for sex and were likely clients of FSWs. These findings, alongside previous evidence 12, 24 suggest that outreach to venues where people meet new sexual partners may be an effective and complementary strategy for reaching multiple groups at risk for HIV infection, including KP. Further programmatically relevant research is needed to examine the effects of implementing venue‐based testing in combination with other modalities deployed on site, such as self‐testing, peer‐led testing referral and index testing services with voluntary partner elicitation.

In our assessment of the treatment cascade, we noted that HIV status awareness fell well short of the first 90, at 34% for FSWs, 26% for MSM and 32% for TGW. However, both treatment uptake and subsequent viral suppression appear relatively high among individuals who knew their status prior to the survey. In fact, we saw that four in five MSM and FSWs on treatment had suppressed VL, which is even higher than the 67.6% reported by a study of 2313 patients on ART at five hospitals around Port‐au‐Prince 25. Thus, intensifying efforts to achieve the first step of the cascade could be an effective strategy for accelerating progress towards 90‐90‐90 targets among MSM and FSW. Further attention should be paid to supporting TGW to sustain treatment for HIV, as we found that only 46% of TGW on treatment (i.e. six of thirteen) were virally suppressed. Further implementation and patient‐oriented research may help determine whether and why treatment outcomes are poorer among TGW.

The high proportion of participants with a positive syphilis test indicates that syphilis infection may also be common among KP, especially FSWs and TGW. HIV prevalence was much higher among individuals with syphilis compared to those without it, reinforcing the need for integrated screening and treatment of STIs as part of a basic package of KP health services. This result is consistent with a survey from the Dominican Republic reporting that the odds of HIV among MSM and TGW with a positive syphilis test was 4.9 (95% CI 2.5, 9.7) times the odds among those with a negative syphilis test 22. STI screening and treatment may serve as an important entry point for comprehensive HIV prevention, treatment and care services for KP, and vice versa.

While access to certain prevention services, such as condoms and HTS, appears to be widespread, other services, including access to lubricant and STI screening and treatment, are lacking among KP. Structural barriers such as homelessness, food insecurity and economic instability are likely important impediments, and the pervasiveness of these barriers in our study population argues for structural interventions to improve the basic socio‐economic wellbeing of KP in Haiti. Interventions to improve access to health services or promote behaviour change are likely to be ineffective if they fail to address the underlying social determinants of health 26. In our study, social vulnerabilities such as food insecurity and economic instability were common among KP. While the confidence intervals were wide, economic instability was nonetheless significantly associated with HIV among MSM and food insecurity and economic instability were significantly associated with HIV among TGW. Moreover, our findings suggest that exposure to discrimination and its psychosocial and physical consequences and correlates, including IPV, rape and imprisonment, exacerbate the risk of HIV infection among KP. These results are consistent with previous research conducted among KP in the Caribbean region 21, 27, 28, 29, 30, 31, 32. For example, a 2015 survey of 556 MSM and 137 TGW in Jamaica found that 48% of MSM and 60% of TGW were food insecure and one‐third of MSM and half of TGW had unstable housing 29. Both food insecurity and unstable housing were associated with higher odds of police harassment, and homelessness was associated with HIV infection among TGW 28. Nearly half of TGW in Jamaica had ever been raped, and history of rape was associated with higher odds of HIV infection. Incarceration was associated with reduced odds of HIV testing among TGW 28 and increased odds of HIV infection among MSM 21.

Not only do socio‐economic vulnerability and structural barriers increase HIV risk, they also affect care retention and quality of life for KP living with HIV. Recent findings from the region suggest that inability to pay for care, lack of food and shelter, and depression due to lack of employment opportunities conspire to interrupt HIV care and adversely affect the health of KP 23. Qualitative data from the Dominican Republic indicate that lack of money for transportation, insufficient food and IPV are major barriers preventing FSWs living with HIV from adhering to ART 33, 34. Our findings support and extend evidence from the region that structural factors are associated with HIV among KP. Further research is needed to estimate the causal effects of these factors and tease out the actionable pathways to more proximate determinants of HIV acquisition, treatment outcomes and onward transmission.

It is worth noting that homelessness appeared to protect against HIV for MSM and FSWs, although this result may be due to collinearity with other characteristics such as socio‐economic status. For example, we observed higher HIV prevalence among MSM with greater economic instability (Table 2) and higher educational attainment (Table S2). Controlling for these factors resulted in a non‐significant association between homelessness and HIV among MSM and a marginally significant association among FSWs.

While our findings are based on objective biomarker and survey data derived from a study sample that was generally representative of KP in Haiti, our study had several limitations. The overall study refusal rate was 9.2%, comparable to that of the last Integrated Bio‐Behavioural Survey (IBBS) in Haiti, which reported refusal rates of 8% for FSWs and 15% for MSM 7, 8. Sampling weights were adjusted to account for predicted probabilities of refusal, but it is possible that some individuals declined to participate in interviews and testing because they already knew they were HIV positive. However, we performed a sensitivity analysis and found that HIV prevalence among FSWs would be 8.3% if all individuals who declined to participate were, in fact, HIV‐positive FSWs – only 0.6% points higher than our estimate of 7.7%. In addition, it was particularly challenging to recruit MSM, regardless of testing history, due to the stigma faced by the LGBTQ community in Haiti and a specific event that received national attention in September 2016 35. Notwithstanding these difficulties, we find that our HIV prevalence estimate among MSM is consistent with that reported by the 2014 IBBS when we restrict our data to construct a comparable study population that is (1) exclude participants from geographical departments not covered by the IBBS or between ages 15 and 17 years, and (2) group together MSM and TGW.

Among participants who were virally suppressed, 70% did not report being on ART. This could point to possible problems with the blood samples or to participants failing to disclose that they were positive and on treatment for fear of discrimination. Although we followed best practices for sample collection and storage, we did not test samples for denaturization. We also did not test for drug metabolites, limiting our ability to comment on participant non‐disclosure. Due to these possibilities, as well as the small numbers of participants with HIV, cascade estimates should be interpreted with caution. These limitations notwithstanding, intensified peer and other patient‐level supports will be needed to improve retention in care and viral suppression to reach the second and third 90s.

Another limitation of our study is that we recruited participants solely from public venues. Consequently, the SEs we present are not total population denominators. They are, however, higher than previous estimates – with the exception of those derived from the unique object multiplier method, which suffered from methodological challenges 7, 8 – and are comparable to recent SEs for the Dominican Republic 6. Nevertheless, it is important that efforts to reach KP do not overlook more hidden sub‐populations who may only socialize in private or only meet partners online. Finally, the small number of TGW study participants limits the precision of our estimates and therefore the conclusions we can draw about TGW in Haiti. We were not able to calculate SEs for TGW, but based on the typical perceptions of venue informants it is likely that TGW are included in the SEs for MSM.

## 5 Conclusions

We demonstrate a continued need for targeted efforts to prevent and treat HIV among KP in Haiti. Based on our findings, we recommend that peer navigators are involved in outreach interventions to reach KP living with HIV who are unaware of their status through venue‐based and other targeted testing modalities, coupled with facilitated linkage to care and treatment. Systematic venue mapping at the community and department levels can identify priority areas for outreach‐based service delivery. In addition, given the high prevalence of syphilis observed, STI screening and treatment should be better integrated into service delivery packages for all KP. Given the observed associations between structural and psychosocial barriers and HIV, KP would likely benefit from the following additional services: IPV and social vulnerability screening, intensified case management and referral to KP‐friendly service providers trained in case management and structural violence mitigation. Finally, future research and KP programming should pay special attention to the high burden of HIV among TGW.

## Competing interests

The authors have no competing interests to declare.

## Authors’ contributions

SW, MH and LZ conceived and designed the study. LZ and JM managed the data collection. LZ and JE analysed the data, and SW, MH and JM assisted in interpretation. LZ drafted the article and all authors critically reviewed and approved the final manuscript.

## Supporting information


**Figure S1.** Map of 2339 validated venues where people meet new sexual partners by KP, PLACE 2016

**Table S1.** Characteristics of 2339 validated venues where people meet new sexual partners across 10 geographical departments, PLACE 2016
**Table S2.** Number and per cent of participants who tested positive for HIV by age group and education level, PLACE 2016
**Table S3.** Number and per cent of participants at each step of the HIV treatment cascade by KP, PLACE 2016
**Table S4.** Key population size estimates by geographical department, PLACE 2016

**Data S1.** Survey Instrument
