# Supplementary figures and images for: The burden of HIV among female sex workers, men who have sex with men and transgender women in Haiti: results from the 2016 Priorities for Local AIDS Control Efforts (PLACE) study
Source: J Int AIDS Soc. 2019 Jul 9;22(7):e25281. doi: 10.1002/jia2.25281 (PMC6615490; doi:10.1002/jia2.25281)

Supplemental Figure. Map of 2,339 validated venues where people meet new sexual partners by KP, PLACE 2016
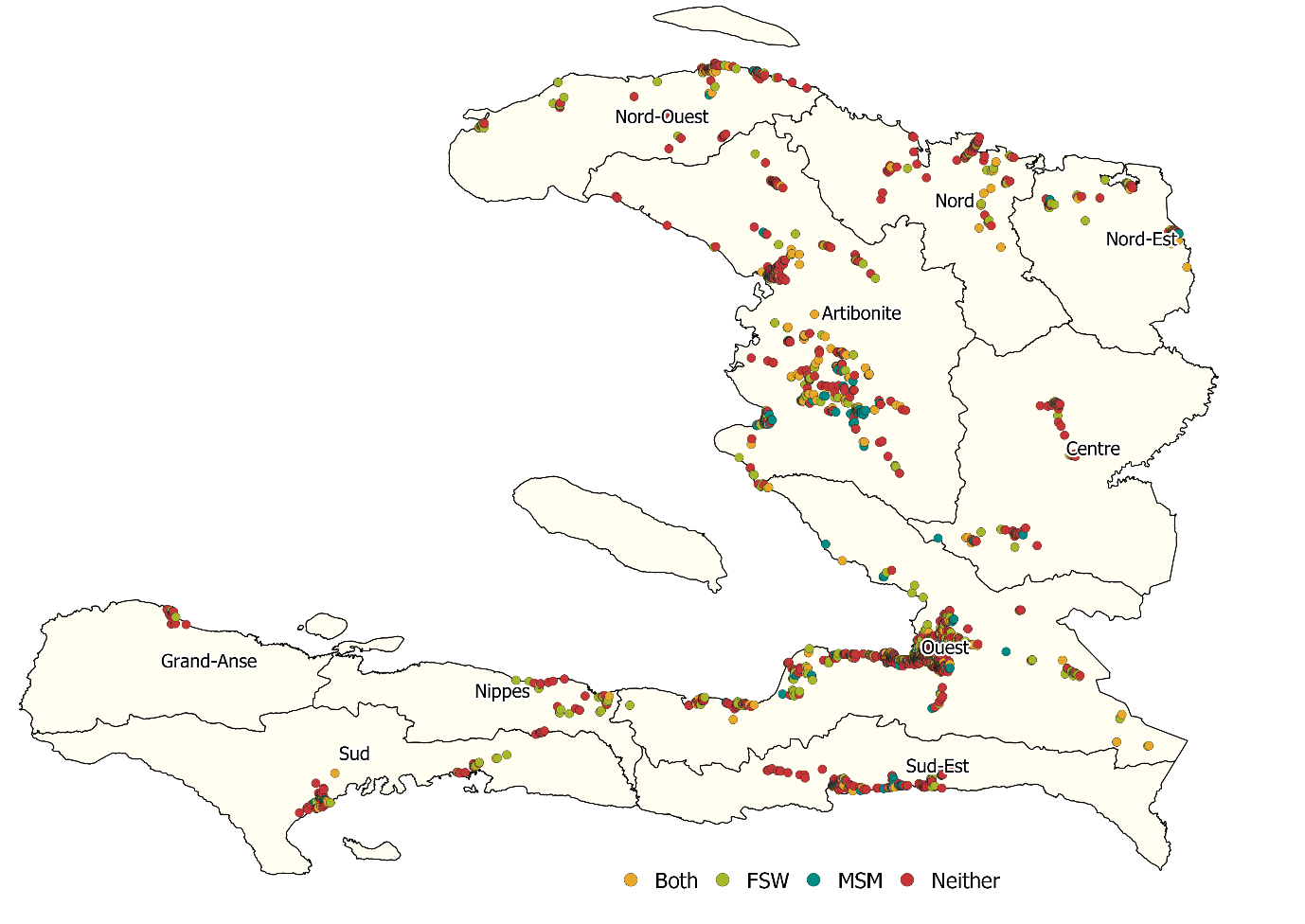

Supplement: Supplementary file 1 — Figure S1. Map of 2339 validated venues where people meet new sexual partners by KP, PLACE 2016 [file JIA2-22-e25281-s001.docx]
