# Supplementary material for: The burden of HIV among female sex workers, men who have sex with men and transgender women in Haiti: results from the 2016 Priorities for Local AIDS Control Efforts (PLACE) study
Source: J Int AIDS Soc. 2019 Jul 9;22(7):e25281. doi: 10.1002/jia2.25281 (PMC6615490; doi:10.1002/jia2.25281)
Supplement: Supplementary file 2 — Table S1. Characteristics of 2339 validated venues where people meet new sexual partners across 10 geographical departments, PLACE 2016 Table S2. Number and per cent of participants who tested positive for HIV by age group and education level, PLACE 2016 Table S3. Number and per cent of participants at each step of the HIV treatment cascade by KP, PLACE 2016 Table S4. Key population size estimates by geographical department, PLACE 2016 [file JIA2-22-e25281-s002.docx]

Supplemental Table 1. Characteristics of 2,339 validated venues where people meet new sexual partners across 10 geographic departments, PLACE 2016

|  | Proportion of Venues | *n* |  | Proportion of Venues | *n* |
| --- | --- | --- | --- | --- | --- |
| Department |  |  | **Amenities and Activities** |  |  |
| Artibonite | 23.0 | *537* | Electricity | 72.2 | *1,673* |
| Centre | 3.9 | *90* | Tap Water | 45.9 | *1,064* |
| Grande-Anse | 1.0 | *23* | Indoor Toilet | 67.2 | *1,557* |
| Nippes | 2.1 | *49* | Beds on Site | 47.3 | *1,095* |
| Nord | 4.7 | *110* | KP Living on Site | 13.2 | *306* |
| Nord-Est | 4.9 | *115* | Sex on Site | 56.8 | *1,319* |
| Nord-Ouest | 6.5 | *152* |  |  |  |
| Ouest | 42.8 | *1,001* |  |  |  |
| Sud | 3.9 | *92* |  |  |  |
| Sud-Est | 7.3 | *170* |  |  |  |
|  |  |  |  |  |  |
| Type of Venue |  |  | **Prevention Services Available in Past 6 Mos.** |  |  |
| Hotel | 22.7 | *530* | Free Male Condoms | 33.0 | *768* |
| Nightclub | 20.4 | *478* | Free Female Condoms | 1.9 | *45* |
| Bar | 18.0 | *421* | Free Lubricant | 8.4 | *194* |
| Brothel | 9.2 | *215* | Visit by Outreach Worker | 15.0 | *348* |
| Baz, Cartel | 5.4 | *127* | HIV Testing on Site | 9.9 | *230* |
| Beach | 4.7 | *109* | Condom Promotion Posters | 1.7 | *40* |
| Street | 3.6 | *84* |  |  |  |
| Cultural, Religious Event | 3.0 | *69* |  |  |  |
| Public Festival | 2.2 | *51* | **Frequented by** |  |  |
| Park, Market | 1.3 | *31* | FSWs | 38.3 | *891* |
| Other | 9.6 | *224* | MSM | 22.2 | *519* |

Supplemental Table 2. Number and percent of participants who tested positive for HIV by age group and education level, PLACE 2016

|  | FSWs | |  | MSM | |  | TGW | |  | Other Men | |  | Other Women | |
| --- | --- | --- | --- | --- | --- | --- | --- | --- | --- | --- | --- | --- | --- | --- |
|  | No. HIV Positive | *%* |  | No. HIV Positive | *%* |  | No. HIV Positive | *%* |  | No. HIV Positive | *%* |  | No. HIV Positive | *%* |
| Age Group |  |  |  |  |  |  |  |  |  |  |  |  |  |  |
| 15 to 19 | 7/77 | *9.1* |  | 2/164 | *1.2* |  | 6/15 | *40.0* |  | 0/70 | *0* |  | 0/30 | *0* |
| 20 to 24 | 22/253 | *8.7* |  | 10/182 | *5.5* |  | 15/42 | *35.7* |  | 7/126 | *5.6* |  | 1/42 | *2.4* |
| 25 to 29 | 27/316 | *8.5* |  | 5/89 | *5.6* |  | 14/22 | *63.6* |  | 4/87 | *4.6* |  | 1/25 | *4.0* |
| 30 to 34 | 16/189 | *8.5* |  | 3/26 | *11.5* |  | 10/12 | *83.3* |  | 2/49 | *4.1* |  | 0/15 | *0* |
| 35 to 39 | 9/80 | *11.3* |  | 1/13 | *7.7* |  | 4/7 | *57.1* |  | 1/25 | *4.0* |  | 1/3 | *33.3* |
| 40 to 44 | 6/29 | *20.7* |  | 2/7 | *28.6* |  | 2/4 | *50.0* |  | 2/17 | *11.8* |  | 0/1 | *0* |
| 45 to 49 | 1/12 | *8.3* |  | 0/5 | *0* |  | 2/2 | *100* |  | 0/4 | *0* |  | 0/1 | *0* |
| 50 to 69 | 0/2 | *0* |  | 0/2 | *0* |  | 0/0 | *0* |  | 0/7 | *0* |  | 0/0 | *0* |
| Education Level |  |  |  |  |  |  |  |  |  |  |  |  |  |  |
| None | 17/52 | *32.7* |  | 0/6 | *0* |  | 0/1 | *0* |  | 0/4 | *0* |  | 0/0 | *0* |
| Primary | 28/221 | *12.7* |  | 2/64 | *3.1* |  | 5/8 | *62.5* |  | 3/44 | *6.8* |  | 2/14 | *14.3* |
| Secondary or Higher | 43/685 | *6.3* |  | 21/418 | *5.0* |  | 48/95 | *50.5* |  | 13/337 | *3.9* |  | 1/103 | *1.0* |

Supplemental Table 3. Number and percent of participants at each step of the HIV treatment cascade by KP, PLACE 2016

|  | FSWs | |  | MSM | |  | | TGW | | |  | | Other Men | | | |  | | Other Women | | | |  |
| --- | --- | --- | --- | --- | --- | --- | --- | --- | --- | --- | --- | --- | --- | --- | --- | --- | --- | --- | --- | --- | --- | --- | --- |
|  | Number | *%* |  | Number | *%* |  | | Number | | *%* | |  | | Number | | *%* | |  | | Number | | *%* | |
| HIV+, Of Those Tested | 88/958 | 9.2 |  | 23/488 | 4.7 | |  | 53/104 | 51.0 | |  | | 16/385 | | 4.2 | |  | | 3/117 | | 2.6 | |  |
| Know Status, Of Those HIV+ | 30/88 | 34.1 |  | 6/23 | 26.1 | |  | 17/53 | 32.1 | |  | | 3/16 | | 18.8 | |  | | 0/3 | | 0 | |  |
| Currently on Treatment, Of Those HIV+ Who Know Status | 21/30 | 70.0 |  | 5/6 | 83.3 | |  | 13/17 | 76.5 | |  | | 2/3 | | 66.7 | |  | | 0/0 | | 0 | |  |
| Virally Suppressed*, Of Those Known to be HIV+ and on Treatment | 17/21 | 81.0 |  | 4/5 | 80.0 | |  | 6/13 | 46.2 | |  | | 2/2 | | 100 | |  | | 0/0 | | 0 | |  |

* Undetectable viral load defined as fewer than 1,360 copies per mL.

Supplemental Table 4. Key population size estimates by geographic department, PLACE 2016

| Department | FSW | MSM |
| --- | --- | --- |
| Artibonite | 12,000 | 8,500 |
| Centre | 2,300 | 2,300 |
| Grande-Anse | 1,200 | 3,300 |
| Nippes | 2,700 | 3,100 |
| Nord | 3,400 | 3,200 |
| Nord-Est | 2,500 | 3,300 |
| Nord-Ouest | 1,300 | 800 |
| Ouest | 9,100 | 7,800 |
| Sud | 3,600 | 3,700 |
| Sud-Est | 2,300 | 2,300 |
| *Total* | 40,400 | 38,300 |
