# Supplementary material for: The burden of HIV among female sex workers, men who have sex with men and transgender women in Haiti: results from the 2016 Priorities for Local AIDS Control Efforts (PLACE) study
Source: J Int AIDS Soc. 2019 Jul 9;22(7):e25281. doi: 10.1002/jia2.25281 (PMC6615490; doi:10.1002/jia2.25281)
Supplement: Supplementary file 3 — Data S1. Survey Instrument [file JIA2-22-e25281-s003.docx]

| Date: | | ______/______/______ |
| --- | --- | --- |
| C1 | Interviewer: |  |
| C2 | Hot Spot ID: |  |
| C3 | Hot Spot Name: |  |
| C4 | Department: |  |
| C5 | Arrondisement: |  |
| C6 | Commune: |  |

| **RECRUITMENT OF RESPONDENTS** | | |
| --- | --- | --- |
| **READ:** Hello. My name is _______. I am working with LINKAGES, a project that is working with the Ministry of Health to improve HIV prevention and treatment programs in Haiti. We are currently conducting a research study to better understand what the needs are at the community level regarding the prevention of HIV. I would like to give you a description of this study, and then you can decide if you want to participate. You must be at least 15 years old to participate, and you must be willing to give two drops of blood to be tested for syphilis and HIV. If you decide to participate, your responses will be anonymous. Your name will not be linked to your responses, and no one will know that you participated in this study. | | |
| C7 | Are you willing to answer the questions I will ask you and give two drops of blood to be tested for syphilis and HIV? | Yes 1  No 2 |
| C7A | <IF C7=2> Why do you prefer not to participate? |  |
| C7B | <IF C7A=1> Where did you already participate? |  |
| C7C | <IF C7A=5> Other Reason: |  |
| IF RESPONDENT DECLINES TO PARTICIPATE, END THE INTERVIEW. THIS PERSON DOES NOT NEED TO BE TESTED, BUT MAY BE TESTED AT THEIR REQUEST. | | |
| C8 | **INTERVIEWER**: How many people avoided talking to you before this person agreed to participate? In other words, how many people declined to participate but did not answer question C7? |  |

| **DEMOGRAPHICS & SCREENING** | | |
| --- | --- | --- |
| C10 | **INTERVIEWER**: What is the sex of the respondent? | Male 1  Female 2 |
| C11 | What is your age? | Age in Years: |
| C12 | Do you live in this department? | Yes 1  No 2 |
| C72 | <IF C12=2> What department do you live in? |  |
| C71 | Do you live in this arrondisement? | Yes 1  No 2 |
| C73 | <IF C71=2> What arrondisement do you live in? |  |
| C13 | Do you live in this commune? | Yes 1  No 2 |
| C74 | <IF C13=2> What commune do you live in? |  |
| C14 | What is your current relationship status? | Married/Cohabitating 1  In a Relationship 2  Not in a Relationship 3 |
| C15 | What level of education have you completed? | None 0  Primary 1  6ème 2  5ème 3  4ème 4  3ème 5  2ème 6  Rheto 7  Philo 8  University 9 |
| C16 | Are you currently working? | Yes 1  No 2 |
| C17 | <IF C16=1> What type of work do you do? | State Employee 1  Private Sector Employee 2  Construction Worker 3  Farmer 4  Security 5  Military/Police 6  Musician/Dancer 7  Driver 8  Vendor 10  Outreach Worker 11  Health Worker 12  Domestic Worker 13  Bartender 14  Sex Worker 15  Other 88 |
| C18 | <IF C17=88> Other Type of Work |  |

| **READ***:* Now I am going to read you a list of behaviors. You do not have to tell me which of these behaviors you did. Just tell me if you have done any of them in the past three months. | | |
| --- | --- | --- |
| SCREEN | In the past 3 months, have you:  1) had three or more sexual partners,  2) had anal sex with anyone, OR  3) had sex with someone you met at a public festival? | Yes 1  No 2 |
| IF NO TO ALL: THANK THE RESPONDENT, END THE INTERVIEW. THIS PERSON DOES NOT NEED TO BE TESTED, BUT MAY BE TESTED AT THEIR REQUEST. | | |

| **SITE VISITING & RISK BEHAVIORS** | | | | | | | | |
| --- | --- | --- | --- | --- | --- | --- | --- | --- |
| READ: Now I am going to ask you some questions about how often you come here. | | | | | | | | |
| C19 | | How frequently do you come to this site? | Daily 1  4-6 Times Per Week 2  2-3 Times Per Week 3  Once a Week 4  2-3 Times Per Month 5  Once a Month 6  Less Than Once a Month 7  This is My First Time Here 8 | | | | | |
| C19 | | Before today, when is the last time you came here? How many days ago? | | |  | | | |
| C20 | | Besides this place, how many other places have you already gone today to socialize, drink alcohol, or look for a person to have sex with? | | |  | | | |
| C21 | | How many other places do you plan to go to today to socialize, drink alcohol, or look for a sexual partner? | | |  | | | |
| C22 | | Now I am going to ask you about places you went to socialize, drink alcohol, or look for a sexual partner in the past week. For example, did you go out yesterday? | | | Yes 1  No 2 | | | |
| C22A | | <IF C22=1> Where did you go?  **INTERVIEWER:** *Prompt the respondent to mention each place he or she went to socialize, drink alcohol, or look for a sexual partner.* | | |  | | | |
| C23 | | What about the day before yesterday? Did you go anywhere to socialize, drink alcohol, or look for a sexual partner? | | | Yes 1  No 2 | | | |
| 23A | | <IF C23=1> Where did you go?  **INTERVIEWER:** *Prompt the respondent to mention each place he or she went to socialize, drink alcohol, or look for a sexual partner.* | | |  | | | |
| C24 | | What about on [three days ago]? Did you go anywhere to socialize, drink alcohol, or look for a sexual partner? | | | Yes 1  No 2 | | | |
| C24A | | <IF C24=1> Where did you go?  **INTERVIEWER:** *Prompt the respondent to mention each place he or she went to socialize, drink alcohol, or look for a sexual partner yesterday.* | | |  | | | |
| C25 | | What about on [four days ago]? Did you go anywhere to socialize, drink alcohol, or look for a sexual partner? | | | Yes 1  No 2 | | | |
| C25A | | <IF C25=1> Where did you go?  **INTERVIEWER:** *Prompt the respondent to mention each place he or she went to socialize, drink alcohol, or look for a sexual partner yesterday.* | | |  | | | |
| C26 | | What about on [five days ago]? Did you go anywhere to socialize, drink alcohol, or look for a sexual partner? | | | Yes 1  No 2 | | | |
| C26A | | <IF C26=1> Where did you go?  **INTERVIEWER:** *Prompt the respondent to mention each place he or she went to socialize, drink alcohol, or look for a sexual partner yesterday.* | | |  | | | |
| C27 | | What about on [six days ago]? Did you go anywhere to socialize, drink alcohol, or look for a sexual partner? | | | Yes 1  No 2 | | | |
| C27A | | <IF C27=1> Where did you go?  **INTERVIEWER:** *Prompt the respondent to mention each place he or she went to socialize, drink alcohol, or look for a sexual partner yesterday.* | | |  | | | |
| **READ**: Now I am going to ask you a few questions about your sexual behaviors. Remember that all your answers are confidential. | | | | | | | | |
| C28 | | How old were you the first time you had sex? | |  | | | | |
| C29 | | In the past 12 months, have you had sex with men, women, or both? | | Men 1  Women 2  Both 3 | | | | |
| C30 | | <IF C29=1 or 3> In the past four weeks, how many men have you had sex with? | |  | | | | |
| C30A | | <IF C29=1 or 3> How many of these [C30] men were men you had sex with for the first time in the past four weeks? | |  | | | | |
| C31 | | <IF C29=2 or 3> In the past four weeks, how many women have you had sex with? | |  | | | | |
| C31A | | <IF C29=2 or 3> How many of these [C31] women were women you had sex with for the first time in the past four weeks? | |  | | | | |
| C32 | | Think about all the people you had sex with in the past 12 months. In total, how many different people did you have sex with in the past 12 months? | |  | | | | |
| C33 | | Do you currently have a primary sexual partner, such as a husband or wife, boyfriend or girlfriend, someone you live with, or another primary partner? | | Yes 1  No 2 | | | | |
| C34 | | Sometimes people receive money in exchange for sex. Have you ever received money in exchange for sex? | | Yes 1  No 2 | | | | |
| C34A | | <IF C34=1> When was the last time you received money in exchange for sex? | | Past 30 Days 1  2-3 Months Ago 2  4-6 Months Ago 3  6-12 Months Ago 4  > 1 Year Ago 5 | | | | |
| C34B | | <IF C34=1> The last time you received money in exchange for sex, did you use a condom? | | Yes 1  No 2 | | | | |
| C34C | | <IF C34=1> How old were you the first time you received money in exchange for sex? | | AGE IN YEARS: | | | | |
| C35 | | Sometimes people receive gifts or favors in exchange for sex, such as phones, jewelry, clothes, drinks, or transportation. Have you ever received gifts or favors in exchange for sex? | | Yes 1  No 2 | | | | |
| C35A | | <IF C35=1> When was the last time you received gifts or favors in exchange for sex? | | Past 30 Days 1  2-3 Months Ago 2  4-6 Months Ago 3  6-12 Months Ago 4  > 1 Year Ago 5 | | | | |
| C35B | | <IF C35=1> The last time you received gifts or favors in exchange for sex, did you use a condom? | | Yes 1  No 2 | | | | |
| C36 | | Sometimes people receive economic assistance in exchange for sex, such as help paying the rent, utility bills, or school fees. Have you ever received economic assistance in exchange for sex? | | Yes 1  No 2 | | | | |
| C36A | | <IF C36=1> When was the last time you received economic assistance in exchange for sex? | | Past 30 Days 1  2-3 Months Ago 2  4-6 Months Ago 3  6-12 Months Ago 4  > 1 Year Ago 5 | | | | |
| C36B | | <IF C36=1> The last time you received economic assistance in exchange for sex, did you use a condom? | | Yes 1  No 2 | | | | |
| C37 | | <IF C34=1, C35=1 or C36=1> Has anyone ever talked to you about the legal rights that people who receive money or gifts in exchange for sex have? | | Yes 1  No 2 | | | | |
| C37A | | <IF C37=1> Who talked to you about that? | |  | | | | |
| C38 | | In the past twelve months, have you paid anyone to have sex with you? | | Yes 1  No 2 | | | | |
| C38A | | <IF C38=1> The last time you paid someone to have sex with you, did you use a condom? | | Yes 1  No 2 | | | | |
| C39 | | Have you ever given someone a gift in exchange for sex, such as a phone, jewelry, clothes, drinks, or any type of economic assistance? | | Yes 1  No 2 | | | | |
| **ACCESS TO SERVICES** | | | | | | | |  |
| **READ**: Now I am going to ask you a few questions about the health services that are available in this area. | | | | | | | | |
| C40 | | Do you know where to go to get tested for HIV? | | | | | Yes 1  No 2 |  |
| C40A | | <IF C40=1> Where? | | | | |  |  |
| **READ**: In the past twelve months, have you received information about HIV/AIDS… | | | | | | | |  |
| C41A | | …from a peer educator or community health worker? | | | | | Yes 1  No 2 |  |
| C41B | | …at a clinic, hospital or pharmacy? | | | | | Yes 1  No 2 |  |
| C41C | | …on the radio? | | | | | Yes 1  No 2 |  |
| C41D | | …from a friend or family member? | | | | | Yes 1  No 2 |  |
| **READ**: Can any of the following actions transmit HIV? | | | | | | | |  |
| C42A | | Drinking from the same glass as someone infected with HIV. | | | | | Yes 1  No 2 |  |
| C42B | | Shaking hands with someone infected with HIV. | | | | | Yes 1  No 2 |  |
| C42C | | Having sex without a condom with someone infected with HIV. | | | | | Yes 1  No 2 |  |
| C42D | | Hugging someone infected with HIV. | | | | | Yes 1  No 2 |  |
| C43 | | What do you think are the chances that you might become infected with HIV? | | | | | Impossible 1  Low 2  Moderate 3  High 4  Do Not Know 5  NA (Already Infected) 6 |  |
| **READ**: Now I am going to ask you a few questions about the availability of condoms and lubricant in this area. | | | | | | | |  |
| C44 | | Have you received any condoms for free in the past 12 months? | | | | | Yes 1  No 2 |  |
| C45 | | Have you received any lubricant for free in the past 12 months? | | | | | Yes 1  No 2 |  |
| C46 | | Have you purchased any condoms in the past 12 months? | | | | | Yes 1  No 2 |  |
| C47 | | Have you purchased any lubricant in the past 12 months? | | | | | Yes 1  No 2 |  |
| C48 | | If you wanted a condom, would it be difficult to find one quickly? | | | | | Yes 1  No 2 |  |
| C49 | | If you wanted lubricant, would it be difficult to find some quickly? | | | | | Yes 1  No 2 |  |
| C51 | | <IF C10=2> Besides condoms, do you use any type of family planning? | | | | | No 0  Yes, Pills 1  Yes, Injection 2  Yes, IUD 3  Yes, Implant 4  Yes, Other 5 |  |
| C52 | | <IF C50=5> Other Method: | | | | |  |  |
| C53 | | <IF C50>0> Where do you obtain your family planning? | | | | |  |  |
| C54 | | The last time you had vaginal sex, did you use a condom? | | | | | Yes 1  No 2  Never Had Vaginal Sex 3 |  |
| C55 | | The last time you had anal sex, did you use a condom? | | | | | Yes 1  No 2  Never Had Anal Sex 3 |  |
| C56 | | The last time you had anal sex, did you use lubricant? | | | | | Yes 1  No 2  Never Had Anal Sex 3 |  |

| **STIGMA AND VULNERABILITY** | | |
| --- | --- | --- |
| **READ:** Now I am going to ask you a few questions about the problems people face in their lives. | | |
| C57 | In the past 12 months, have you always had enough food to eat? | Yes 1  No 2 |
| C58 | In the past 12 months, have you always had enough money to support yourself? | Yes 1  No 2 |
| C59 | In the past 12 months, have you ever been homeless? | Yes 1  No 2 |
| C60 | In the past 12 months, have you ever been mistreated by a health care worker? Or have you avoided seeking health care because you knew you would not be received well on account of who you are or the work that you do? | Yes 1  No 2 |
| C61 | In the past 12 months, have you been the victim of any physical violence? | Yes 1  No 2 |
| C62 | In the past 12 months, have you suffered any physical violence from a sexual partner, spouse, boyfriend, or girlfriend? | Yes 1  No 2 |
| C63 | Have you ever been forced to have sex against your will? | Yes 1  No 2 |
| C64 | Have you ever been forced to have sex without a condom? | Yes 1  No 2 |
| C65 | Have you ever spent a night in jail or prison? | Yes 1  No 2 |
| C66 | Have you ever been beaten by the police? | Yes 1  No 2 |
| C67 | How many children are you currently taking care of? | Number: |
| C68 | Do you see yourself as a man or as a woman? | Man 1  Woman 2 |
| C69 | Do you identify as LGBT? | Yes 1  No 2 |
| INTERVIEWER: TAKE THE PARTICIPANT TO A NURSE WHO WILL COMPLETE THE SECTION BELOW BEFORE ADMINISTERING PRE-TEST COUNSELING, RAPID TESTING FOR HIV AND SYPHILIS, AND POST-TEST COUNSELING | | |

| **SYMPTOMS AND TEST RESULTS** | | |
| --- | --- | --- |
| THIS SECTION IS ADMINISTERED BY A NURSE | | |
| R1 | In the past 12 months, have you been tested for tuberculosis? | Yes 1  No 2 |
| R2 | <IF R1=1> In the past 12 months, have you been given a diagnosis of tuberculosis? | Yes 1  No 2 |
| R3 | In the past two weeks, have you had a cough, fever, night sweats, or unexplained weight loss? | Yes 1  No 2 |
| R4 | Are you circumcised? | Yes 1  No 2  Do Not Know 3  N/A 8 |
| R5 | Do you currently have an unusual discharge from your penis, vagina, or anus? | Yes 1  No 2 |
| R6 | Do you currently have sores around your penis, vagina, or anus? | Yes 1  No 2 |
| R7 | Before today, have you ever been tested for HIV? | Yes 1  No 2 |
| R8 | <IF R7=1> Before today, when was the last time you were tested for HIV? | Past 3 Months 1  Past 6 Months 2  Past Year 3  > 1 Year Ago 4 |
| R9 | <IF R7=1> Has a health worker ever told you that you have HIV? |  |
| R10 | <IF R9=1> Have you taken any medication for HIV? |  |
| R11 | <IF R10=1> Are you currently taking medication for HIV? |  |
| R12 | <IF R11=1> During the past week, how many days did you take medication for HIV? | 0 Days  1 or 2 Days  3 or 4 Days  5 or 6 Days  Every Day |
| R13 | <IF R10=1> Where do you obtain medication for HIV? |  |
| R14 | ENTER HIV TEST RESULT | Positive 1  Negative 2  Indeterminate 3 |
| R15 | ENTER SYPHILIS TEST RESULT | Positive 1  Negative 2  Indeterminate 3 |
| R16 | <IF R14=1> ENTER HIV CONFIRMATION TEST RESULT | Positive 1  Negative 2  Indeterminate 3  N/A 8 |
| R17 | <IF R14=1> ENTER NUMBER OF DRIED BLOOD SPOTS OBTAINED | Two 2  Three 3  Four 4  Five 5  N/A 8 |
